# A robust privacy-preserving federated framework for kidney CT image classification using transfer learning models

**DOI:** 10.3389/frai.2026.1840721

**Published:** 2026-09-04

**Authors:** Sai Sri Hemantha Konala, Srinivas Koppu

**Affiliations:** School of Computer Science Engineering and Information Systems, Vellore Institute of Technology, Vellore, Tamil Nadu, India

**Keywords:** advanced encryption standard, federated learning, federated weighted averaging, kidney disorders, transfer learning

## Abstract

**Introduction:**

Kidney abnormalities, including cysts, tumors, and stones, are the most common renal disorders that can lead to severe complications such as chronic kidney disease or renal failure. Deep learning-based medical image analysis offers an effective approach for the accurate classification of kidney abnormalities, aiding the early diagnosis of renal disorders. However, its centralized training leads to inadequate privacy protection.

**Methods:**

Considering the importance of ensuring individuals' data privacy, this study proposes a novel federated transfer learning framework for accurate classification of renal abnormalities using 12,446 kidney CT scan images and simultaneously preserves data privacy. CT scan images were preprocessed by resizing and normalization, followed by data augmentation techniques, including random rotations (±30°), horizontal flips, and color jitter, to address class imbalance and improve model generalization. Five pre-trained deep learning models such as MobileNetV2, EfficientNetV2-S, ResNet50, DenseNet121, and InceptionResNetV2 were trained across seven federated clients. Federated weighted averaging was employed for aggregation, and AES-256 encryption in CBC mode was applied to all model parameter transmissions between clients and the server.

**Results:**

MobileNetV2 achieved the best performance, attaining 99.48% accuracy, 99.29% precision, 99.32% recall, 99.3% F1-score, 0.9999 AUC-ROC, and log loss of 0.0247. Cross-client validation produced an average accuracy of 98.85% with a generalization gap of only −0.0063, indicating strong generalization across client datasets.

**Discussion:**

The proposed framework provides an effective balance between privacy preservation and communication efficiency, highlighting its potential for deployment in distributed clinical environments for kidney disease diagnosis.

## 1 Introduction

Globally, over 850 million people have been diagnosed with Chronic Kidney Disease (CKD) and is one of the major leading disease apart from other non-communicable disease such as diabetes, cancer etc. In the near future, it is expected that CKD may surpass other highly prevalent diseases such as HIV and diabetes (Jager et al., 2019). Due to its complex nature, CKD is considered as a silent killer and can affect anyone irrespective of race, age, and gender (Kovesdy, 2022). Kidney disorders results in heart-related complications, and increased risk of mortality (Blum et al., 2025). It is predicted that Kidney diseases will be the fifth leading cause of mortality by 2040 (Foreman et al., 2018). A typical preliminary CKD diagnosis involve determining serum creatinine and human serum albumin levels in blood and urine (Vassalotti et al., 2016; Kalantar-Zadeh et al., 2021). The presence of cysts (fluid-filled sacs), tumors, and stones (concentrated minerals) in the kidneys is of significant clinical importance. Hence, an accurate diagnosis of these conditions and effective treatment management are of prime importance to preserve renal health (Kumar et al., 2024). Kidney CT image classification is challenging because renal abnormalities can exhibit substantial variations in morphology and imaging appearance across patients, increasing the complexity of automated analysis (Kumar et al., 2024; Heller et al., 2019). If detected during early stages of disease, the above-mentioned kidney disorders can be managed and may be reversed (Jha et al., 2019). Due to the limited availability of nephrologists, providing timely and appropriate medical care is challenging even when patients are symptomatic (Jha et al., 2019). Delay in the diagnosis of CKD can lead to kidney failure and other serious consequences. Hence, early detection is crucial for mitigating disease progression (Kalantar-Zadeh et al., 2021).

Medical imaging is crucial for the reliable diagnosis of kidney abnormalities. Various imaging techniques are employed to analyze patients data and identify underlying renal disorders (Kumar et al., 2024). Deep learning (DL) has advanced Image processing by facilitating automated feature extraction and substantially improving the performance of critical tasks such as object detection, segmentation, image classification, and image enhancement (Pimpalkar et al., 2025). DL has shown promising results in identifying anomalies across different medical imaging tasks, supporting its broader use in clinical diagnosis (Heidari et al., 2026). However, maintaining data confidentiality and patient privacy poses substantial challenges in securely accessing healthcare information (Sohan and Basalamah, 2023). Federated Learning (FL) handles these concerns by providing model training directly on servers or decentralized devices that retain local data. Instead of sharing sensitive information, model parameters or updates are only shared with the central server, which in turn improves data security and safeguards patient privacy (Sharma and Kumar, 2023).

This study introduces a novel and secure framework for classifying renal disorders using federated learning integrated with advanced deep learning Models. The key contributions of this study are

- A secure federated learning framework incorporating AES-256 encryption is developed for privacy-preserving kidney abnormality classification
- A Federated Weighted Averaging (FedWAvg) aggregation strategy is developed, in which aggregation weights are determined using local classification accuracy and client dataset size.
- To address class imbalance, rotation and flipping augmentations are applied to minority classes during distributed training.
- Five transfer learning architectures, namely ResNet50, DenseNet121, EfficientNetV2-S, InceptionResNetV2, and MobileNetV2, are systematically evaluated within the FL framework.
- The proposed methodology is further evaluated on an independent lung cancer CT dataset to validate its cross-domain generalizability across different medical imaging domains.

The organization of the paper is as follows. Section 2 presents the related work and background on kidney CT image analysis, and federated learning. Section 3 describes the proposed framework, including image preprocessing, data augmentation, the proposed FL framework, AES-based secure model transmission, the FedWAvg aggregation strategy, and the pre-trained models used for FL. Section 4 presents the experimental setup, performance evaluation, comparative analysis, and external validation results, and Section 5 concludes the paper and discusses limitations and future research directions.

## 2 Literature review and background

Numerous studies have been conducted on kidney CT scan classification using diverse methodologies. Most studies focus on the CT kidney dataset for both development and validation, enabling consistent comparison across different approaches.

Pimpalkar et al. (2025) introduced a deep learning framework for classifying kidney CT scan images into Cyst, Tumor, Normal, and Stone classes using pre-trained transfer learning models including InceptionV3, VGG16, CNNAlexNet, and ResNet50. For precise boundary extraction and object separation, they employed Watershed segmentation and Otsu's binarization. The refinement and selection of feature maps have been done through the Relief method. The proposed approach achieved a classification accuracy of 99.96% using the InceptionV3 model. Obaid et al. (2025) proposed a DL-based multi-class classification approach for classifying cysts, tumors, stones, and normal kidneys using a multisource dataset comprising 27,151 urogram and CT-scan images sourced from five repositories. They implemented 16 convolutional neural network models, including InceptionV3, Densenet, Darknet, Resnet variants, Squeezenet, Googlenet, Vgg16. The Darknet53 model exhibited an accuracy of 99.69% in detecting kidney abnormalities.

Islam et al. (2022) presented a comprehensive AI-driven approach to detect kidney diseases using CT scan images. The dataset, containing 12,446 annotated images, was processed through three CNN-based models (VGG16, ResNet50, Inception v3) and three Vision Transformers (EANet, CCT, Swin Transformer). The Swin Transformer outperformed all models with 99.30% accuracy, while VGG16 demonstrated superior explainability for anatomical abnormalities. The research highlights the importance of explainable AI in medical imaging and suggests future improvements through privacy-preserving learning, hybrid model integration, and real-time clinical deployment. Pande and Agarwal (2024) proposed a novel technique for detecting various kidney abnormalities through a Deep Learning approach. The YOLOv8n-cls model was utilized for multi-class classification, which incorporates a convolutional neural network (CNN) for effective image processing in a single pass. The proposed model obtained an accuracy of 82.52%, precision of 85.76%, F1 score of 75.72%, a specificity of 93.12%, and recall of 75.28%.

Sasikaladevi and Revathi (2024) proposed the use of deep learning techniques for detecting end-stage renal disease from CT scan images. High-order features are extracted using Deep convolutional neural networks (CNNs). A three-layer Hypergraph convolutional neural networks (HGCN) build hyperedges from extracted features to do representational learning. The model is validated using hold-out validation and achieves an accuracy of 99.71%. The Gradient-Weighted Class Activation Mapping (GradCAM) was used to visualize the various renal disorders. Kumar et al. (2024) focused on developing an AI-based technique for classifying renal abnormalities (cysts, stones, and tumors) on 12,446 kidney CT scan images with deep transfer learning. For identification of the region of interest, various Segmentation methods like Otsu's binarization, Watershed transformation, and Distance transform are utilized. Additionally, Contour-based features such as area, perimeter, and aspect ratio were extracted to improve disease recognition. Several deep learning models, including Xception, EfficientNetB0, DenseNet201, InceptionResNetV2, and ResNet50V2, were trained using Adam optimizers, RMSprop, and Stochastic Gradient Descent (SGD). Xception through RMSprop got the highest accuracy (99.89%), while Certain models, including EfficientNetB0 and MobileNetV2 with SGD optimizers and RMSprop, resulted in overfitting issues.

Bhandari et al. (2023) proposed a lightweight customized CNN model for diagnosing kidney abnormalities for effective deployment of IoT devices as well as maintaining the interpretability about model's prediction using LIME and SHAP. The proposed model achieved an accuracy of 99.52% ± 0.84% on test data. Furthermore, an ablation study on a chest X-ray dataset supported the model's explainability in identifying COVID-19, tuberculosis, pneumonia, and healthy cases. Gharahbagh et al. (2024) introduced a deep learning model for the detection and classification of multiple renal disorders using CT images. The proposed method utilizes asymmetric local statistical pixel distributions by splitting input images into non-overlapping windows and constructing histograms of pixel intensities and gradient values using optimally selected asymmetric intervals. The extracted features are fed into a five-layer DL model featuring a Long Short-Term Memory (LSTM) for accurate classification. The proposed model achieved a classification accuracy of 0.9989, precision of 0.984, recall of 0.996, MCC of 0.990, and an F1 score of 0.991.

Dhanabal et al. (2025) developed a CNN-based framework using VGG16 transfer learning to classify CT kidney images, incorporating oversampling and augmentation to address class imbalance. The confusion matrix confirmed reliable classification performance across all four kidney categories. Elbedwehy et al. (2024) introduced a novel hybrid approach that integrates the advanced attention mechanisms of ConvNeXt with the powerful feature extraction of AlexNet for the classification of kidney disorders. A key innovation lies in the integration of spatial features from both networks, further improved by self-attention layers, and a custom optimization technique based on Adam optimizer efficiently boosts classification performance. The proposed model demonstrates 99.85% accuracy, 99.95% recall, 99.89% precision, and 99.83% specificity.

In addition to deep learning studies on the kidney dataset, federated learning has been explored on other datasets to highlight its efficiency in secure and distributed model training.

Batra et al. (2025) proposed a FL framework for kidney disease classification using CT scan images. The study employed EfficientNetB0 and an automated augmentation technique called AutoAlbum to improve data diversity across distributed clients. Using standard FedAvg with four simulated clients, the framework achieved an accuracy of 91.16%, precision of 99.17%, recall of 99.16%, and F1 score of 99.15% on the augmented distributed dataset. Heidari et al. (2023) developed a lung cancer detection framework that combines FL with blockchain technology for secure model training across multiple hospitals using chest CT images. Each hospital trained a local CapsNet model on its own data, and blockchain was used to coordinate the global model updates without exposing raw patient records. The proposed methodology was tested on four datasets and achieved 99.69% accuracy. Bhulakshmi and Rajput (2024) developed a Federated deep learning (FedDL) architecture for classification of diabetic retinopathy. They employed five cutting-edge CNN architectures, including DenseNet201, AlexNet, ResNet, VGGNet19, and EfficientNetB7 on the augmented Diabetic Retinopathy Image Dataset (IDRiDL). The Proposed framework achieved the accuracy rates of 82.07%, 92.19%, 94.66%, 91.81%, and 80.02%, respectively.

Tan et al. (2023) introduced an AI-based FL framework for breast cancer classification using the Digital Database for Screening Mammography (DDSM) dataset. Transfer learning was employed for the extraction of significant features from the region of interest (ROI) for future analysis. The proposed model integrates the Synthetic Minority OverSampling Technique (SMOTE) for addressing data imbalance, and FedAvg-CNN with MobileNet in an FL framework. They achieved an accuracy of 98% with 100% recall and an AUC of 99.804%. Srinivasu et al. (2024) developed FL architecture for privacy-preserving medical image classification to evaluate the efficacy of pre-trained models in a decentralized learning environment. For their study, they used two datasets: the Brain Tumor Detection (BR35H) dataset, which contains brain MRI images, and the SARS-CoV-2 CT scan dataset, which contains lung CT scan images. A Convolutional Neural Network (CNN) with Gray-level Co-occurrence Matrix (GLCM) and local binary patterns (LBP) and EfficientNet were employed as local models. They achieved classification accuracy of 97.4 and 98.8%, and Diagnostic Odds Ratios (DOR) of 1,164.54 and 6,825.17 for MRI and CT scans, respectively. AlSalman et al. (2024) proposed a privacy-preserving framework for breast cancer detection using FL combined with Deep Convolutional Neural Networks (DCNNs) using three publicly available mammography datasets, including VINDR-MAMMO, INBREAST, and Chinese Mammography database (CMMD). They trained DCNNs across decentralized nodes, with Homomorphic Encryption in a federated environment. Experimental results demonstrate a classification accuracy of 98.9% in effectively classifying the breast cancer cases into benign and malignant categories.

Albalawi et al. (2024) proposed a FL and transfer learning framework for brain tumor classification using MRI images. A modified VGG16 model was trained on a dataset of 7,023 MRI images in decentralized environment. The proposed method achieved 98% accuracy while maintaining data privacy across participating clients. Saha et al. (2025) proposed Lung-AttNet, an attention-based CNN integrated with FL for privacy-preserving lung cancer detection on a kaggle lung CT scan dataset containing 1,000 CT images. The methodology combined image preprocessing techniques, including white balancing and CLAHE, with a lightweight global attention module (LGAM) to enhance feature extraction. The proposed approach achieved 91.5% accuracy in a centralized setting and 92% in the FL environment.

Comparative analysis presented in Table 1 identifies several challenges, particularly with respect to privacy preservation, secure communication, model aggregation, and generalizability. These challenges motivates the development of the proposed FL approach.

**Table 1:** Comparative analysis of existing research.

References	Dataset	Methodology	Observation	Results (Accuracy obtained)
Islam et al. (2022)	CT kidney dataset (12,446 images)	CNNs (VGG16, ResNet50, InceptionV3); Transfer models: EANet, CCT, Swin Transformer	Missing multimodal data (e.g., ultrasound, MRI)	99.30%
Bhandari et al. (2023)	CT kidney dataset (12,446 images)	Custom CNN; Explainable AI: SHAP, LIME	Only three layers–may limit feature learning capacity	99.52%
Bingol et al. (2023)	CT kidney dataset (12,446 images)	Hybrid deep learning model (29 layers); Relief method for feature optimization; Wide Neural Network classifier	Centralized approach with no privacy preservation or federated learning support	99.37%
Pande and Agarwal (2024)	CT kidney dataset (12,446 images)	Yolov8n-cls for classification (single-pass CNN)	Restricted performance comparison	82.52%
Gharahbagh et al. (2024)	CT kidney dataset (12,446 images)	Feature extraction based on gradient and histogram; Model: DNN with LSTM	Didn't include texture/time-frequency features; may miss complex patterns	99.89%
Kumar et al. (2024)	CT kidney dataset (12,446 images)	Segmentation: Distance Transform, Watershed, Otsu; Models: Xception, DenseNet201, EfficientNetB0, ResNet50V2, InceptionResNetV2, MobileNetV2	No Explainable AI integration, limiting interpretability	99.89%
Sasikaladevi and Revathi (2024)	CT kidney dataset (12,446 images)	Hypergraph Convolutional Neural Network (HGCN) with three layers	Computationally expensive due to hypergraph construction	99.71%
Elbedwehy et al. (2024)	CT kidney dataset (12,446 images)	Hybrid of AlexNet and ConvNeXt with self-attention; Custom Adam-inspired optimizer	Could integrate Explainable AI for clinical relevance	99.85%
Obaid et al. (2025)	27,145 kidney MRI and CT images from five repositories	16 DL models: Googlenet, AlexNet, DenseNet201, InceptionV3, ResNet variants, Darknet variants, EfficientNetB0, MobileNet, InceptionResNetV2, SqueezeNet, ShuffleNet, VGG16	Limited adaptability to other modalities like ultrasound	99.69%
Pimpalkar et al. (2025)	CT kidney dataset (12,446 images)	Segmentation using Watershed and Otsu's binarization; Models: VGG16, ResNet50, AlexNet, InceptionV3	Sensitive to noise or low-contrast CT images	99.96%
Batra et al. (2025)	CT kidney dataset (12,446 images)	Federated Learning with EfficientNetB0; AutoAlbum augmentation (11 techniques); Standard FedAvg aggregation across four simulated clients	No secure parameter transmission; limiting generalizability	99.16%

### 2.1 Deep learning

Deep Learning (DL) is a branch of ML that employs multi-layered neural networks to enable learning of patterns and extract features from large datasets. DL approaches have shown remarkable progress in medical image analysis, especially in areas like image segmentation, detection, and classification (LeCun et al., 2015). DL models like Convolutional Neural Networks (CNNs) can automatically learn hierarchical features from the data, removing the dependency on manual feature engineering. Moreover, training deep networks from scratch often involves huge amounts of labeled data and high computational capacity. To overcome these challenges, Transfer learning offers a robust solution by utilizing pre-trained models trained on extensive benchmark datasets to be fine-tuned for related tasks (Weiss et al., 2016).

### 2.2 Federated learning

Federated learning (FL) is a decentralized technique in which multiple devices or organizations collaboratively train a shared global model, without exchanging their raw data. Instead of transferring data to a central server, each participant (client) locally trains the model and shares only the updated parameters, such as weights or gradients, with the server (Sohan and Basalamah, 2023).

FL plays a crucial role in medical image classification, as it enables collaborative model training without permitting direct access to sensitive patient data. Conventional centralized techniques raise privacy and regulatory concerns, as it allow collecting and pooling medical images from multiple hospitals or institutions. FL handles these concerns by enabling deep learning models to be trained locally on decentralized datasets, allowing the raw medical images to remain securely on the client side. This approach supports data protection regulations like Health Insurance Portability and Accountability Act (HIPAA) and General Data Protection Regulation (GDPR), and also improves model generalization by using diverse data sources. Recent reviews have highlighted the growing adoption of FL in medical imaging, while also identifying challenges related to data heterogeneity, communication efficiency, and privacy preservation in multi-institutional environments (Ciupek et al., 2026). For critical tasks like identifying tumors, cysts, stones, or other abnormalities in kidney CT scans, FL enables collaborative model training while maintaining patient confidentiality, making it especially suitable for real-world deployment in healthcare applications (Koutsoubis et al., 2025).

### 2.3 Research gap and motivation

Kidney disorders often require early diagnosis for effective treatment. However, concerns regarding data privacy limit the use of centralized deep learning approaches in clinical settings. FL enables decentralized model training while preserving data privacy, but several challenges remain. Existing studies commonly use aggregation methods such as FedAvg, which treat all client updates equally and do not consider local model performance. As a result, valuable information from high-performing clients may not be fully utilized, potentially limiting the effectiveness of the global model. In addition, many FL frameworks do not incorporate secure parameter transmission mechanisms, which may expose model updates to unauthorized access during communication. Class imbalance in kidney CT datasets can further bias model learning toward majority classes, reducing the detection performance for less frequent abnormalities. Moreover, most existing studies are evaluated only on kidney datasets, making it difficult to assess whether the learned models can generalize to different medical tasks and clinical environments.

To address these challenges, this study develops a secure FL framework for kidney abnormality classification. First, a Federated Weighted Averaging (FedWAvg) strategy is introduced to incorporate both local dataset size and validation accuracy during model aggregation, giving greater weight to clients with better local performance. Second, AES-256 encryption is employed to secure parameter transmission between clients and the central server, improving communication security and protecting model updates. Third, data augmentation techniques are applied to address class imbalance and improve the classification of minority classes. Finally, the proposed work is evaluated on an independent lung cancer CT dataset to assess its robustness and generalizability beyond kidney imaging.

## 3 Methodology

### 3.1 Dataset description

The Computed Tomography (CT) scan images of the Kidney dataset (cysts, tumors, stones, or normal) were obtained from Kaggle and originally sourced from the Picture Archiving and Communication System (PACS) of multiple hospitals in Dhaka, Bangladesh. https://www.kaggle.com/datasets/nazmul0087/ct-kidney-dataset-normal-cyst-tumor-and-stone It contains both the axial and coronal cuts from contrast-enhanced and non-contrast scans. The coronal views are vertical slices, like looking straight at the person, while axial views are horizontal slices, like looking down through the body. Each DICOM study corresponds to a single confirmed diagnosis at a time. After removing the patient's identifiable information, the DICOM images were then converted into JPG image format. To ensure the correctness of the dataset, a radiologist and a medical technologist performed further verification. The finalized dataset comprises 12,446 images, categorized as

- Cyst-3,709 images
- Tumor-2,283 images
- Stone-1,377 images
- Normal-5,077 images

### 3.2 Dataset preprocessing

After dataset loading, all the images are resized to 224 × 224 pixels and then normalized to the range [0,1] by dividing each intensity value by 255 to accelerate model convergence and improve training performance. After splitting the dataset into training and test sets, augmentation techniques, including horizontal and vertical flipping, random rotations, and brightness modifications, are applied. This step increases the number of training samples in minor classes that align with the majority class, thus reducing the risk of overfitting and handling class imbalances.

### 3.3 Data augmentation for balancing the training set

The dataset was split into training and test sets using an 80:20 ratio, resulting in 9,956 training images and 2,490 test images. This split ensured sufficient data for model training while maintaining a representative test set for unbiased model evaluation. To address class imbalances in the training dataset comprising 4,061 images in Normal, 2,967 images in Cyst, 1,826 images in Tumor, and 1,101 images in Stone, data augmentation techniques include random rotations (±30 degrees), random horizontal flips and color jittering, were applied to the minority classes to match with the majority class samples (Normal class of 4,061 images).

Image rotation enhances model robustness by randomly rotating input images. In this study, images are randomly rotated within a range of −30° to +30°, allowing the network to learn rotational invariance (Gonzalez, 2009). The geometric transformation applied during rotation is represented in Equation 1.


$$\begin{array}{l} \left[\begin{array}{c} a^{\prime }\\ b^{\prime } \end{array}\right]=\left[\begin{array}{cc} \cos\theta & -\sin\theta \\ \sin\theta & \cos\theta \end{array}\right]\left[\begin{array}{c} a\\ b \end{array}\right],\;\theta \sim \text{Uniform}\left(-30^{{}^{\circ}},+30^{{}^{\circ}}\right). \end{array}$$ (1)

In compact form, it can be expressed as Equation 2.


$$\begin{array}{l} T_{\text{rot},\theta }\left(a\right)=R\left(\theta \right)a. \end{array}$$ (2)

where (*a*′, *b*′) and (*a, b*) indicates the rotated pixel and original coordinates and *R*(θ) is the 2D rotation corresponding to a random angle θ in the range of −30° to +30°.

The horizontal flip transformation mirrors the image along its vertical axis, effectively reversing left and right spatial orientations to enhance spatial invariance. The transformation can be expressed as Equation 3 (Gonzalez, 2009)


$$\begin{array}{l} T_{\text{flip}}\left(x\right)\left(f,g\right)=x\left(W-f,g\right), \end{array}$$ (3)

where *x*(*f, g*) denotes the pixel intensity of spatial coordinates (*f, g*) and *W* is the image width.

Color jittering modifies color brightness, intensity, contrast, and sharpness of the images to enhance visual diversity. The transformation can be expressed as Equation 4 (Gonzalez, 2009):


$$\begin{array}{l} \begin{array}{c} T_{\text{jitter},\alpha ,\beta }\left(y\right)=\alpha y+\beta ,\\ \alpha \sim \text{Uniform}\left(0.7,1.3\right),\\ \beta \sim \text{Uniform}\left(-0.3,0.3\right), \end{array} \end{array}$$ (4)

where α controls the image contrast and β adjusts its brightness.

### 3.4 Proposed federated framework

**Figure 1:**
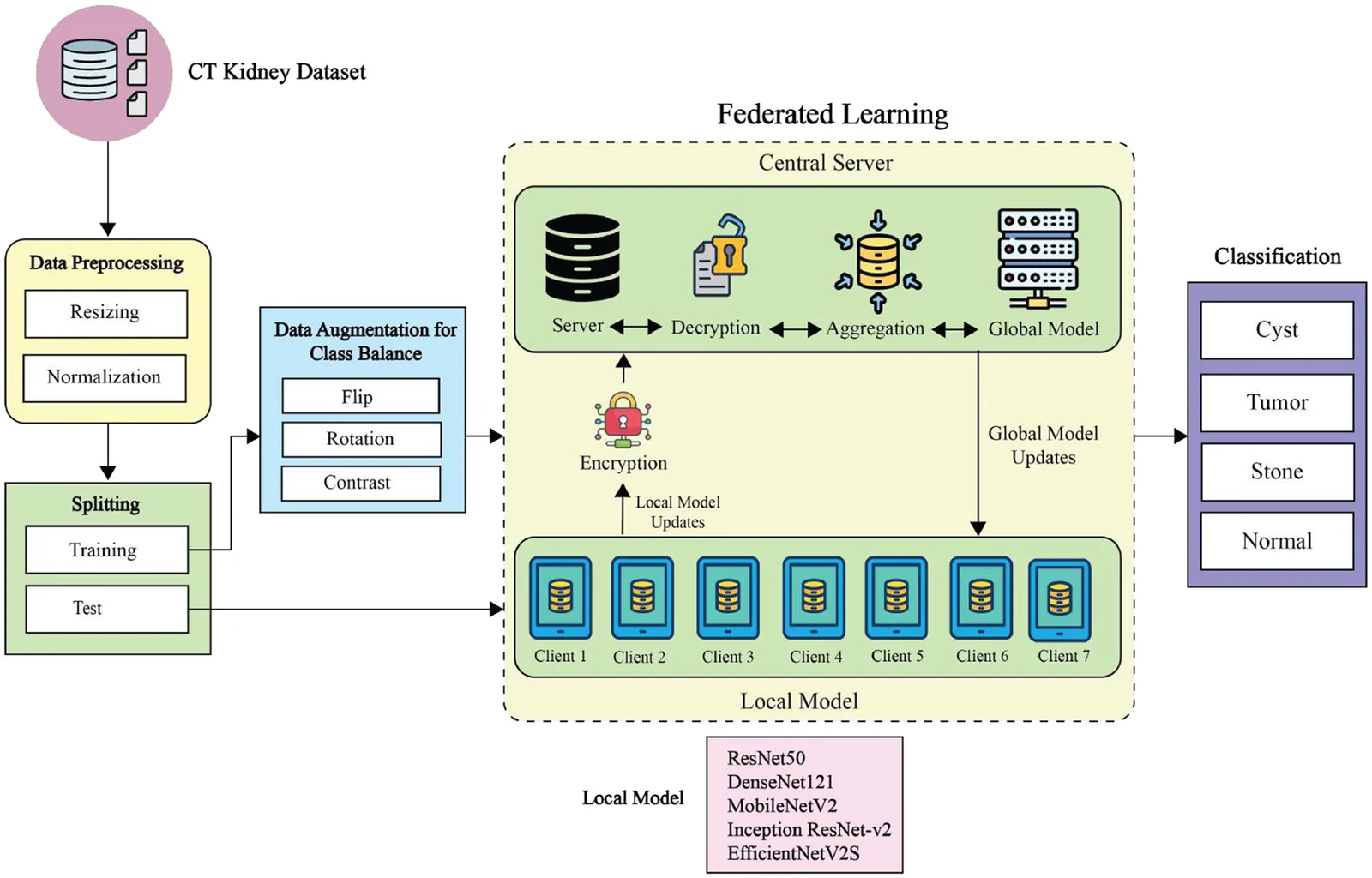
Proposed federated learning framework.

FL ensures privacy by allowing devices to share only model updates, such as weights and gradients. These updates are aggregated by a central server to build a global model without direct access to the local datasets. Clients are devices with local datasets that train the models, while the central server coordinates the process and aggregates the model updates. DL models, including MobileNetV2, EfficientNetV2-S, ResNet50, DenseNet121, and InceptionResNetV2, are trained at the client and server sides. These models are chosen for their optimal trade-off between performance and computational efficiency. The proposed federated learning framework is represented in Figure 1.

The steps involved in the proposed Framework are:

*Input:* CT scan images of renal disorders, including cyst, tumor, stone, and normal classes

*Output:* Optimal Deep Learning model for classification of kidney disorders in an FL environment.

*Model initialization:* Multiple deep learning models, including MobileNetV2, Efficient-NetV2-S, ResNet50, InceptionResNetV2, and DenseNet121, are initialized for their proven track record for handling image classification tasks effectively. These DL models are pre-trained on ImageNet and offer a robust basis for transfer learning, and are adapted for multi-class classification of various kidney disorders.

*Transfer learning strategy:* All five pre-trained models were initialized with ImageNet weights and adapted for the kidney CT classification task using transfer learning. During FL, the pre-trained backbone layers of each model were frozen, while only the final 10 trainable layers and the newly added classification head were fine-tuned on the local client datasets. Furthermore, the original output layer of each model was replaced with a fully connected layer consisting of four neurons corresponding to the target classes (Cyst, Normal, Stone, and Tumor). This transfer learning technique helped the pre-trained models to effectively learn features relevant to kidney CT image classification while reducing the risk of overfitting.

*Federated learning environment:* The proposed framework involves multiple global rounds (1 to P), where three clients are randomly selected from a total of seven participating clients in each round to receive the global model and perform local training on their private datasets for a defined number of epochs. Following local training, each clients encrypt its model weights using the Advanced Encryption Standard (AES-256) in Cipher Block Chaining (CBC) mode to ensure secure transmission to the central server. At the server, these encrypted weights are decrypted and aggregated using the Federated Weighted Averaging (FedWAvg) algorithm, which assigns aggregated weights to each selected client based on both dataset's size and local validation accuracy. The aggregated weights are then utilized by the server to update the global model. This iterative process continues for a fixed number of communication rounds to refine the global model. The experimental setup follows an IID data distribution, as similar class proportions are maintained across all client partitions. Although the data are IID, some variation in local model performance was observed across clients, which supports the use of accuracy-weighted FedWAvg aggregation.

*Client distribution and data allocation:* The balanced training set of 16,244 CT images (4,061 images per class) was partitioned into seven clients using Stratified K-fold (n_splits = 7). This ensured that the class distribution was maintained across all clients, with each client receiving approximately 2,320 imagescontaining similar proportions of Normal, Cyst, Stone, and Tumor cases. Each fold was assigned to a separate client for local training during FL. During each communication round, three clients were randomly selected to participate in local training.

*Model evaluation and model selection:* Standard evaluation metrics including Accuracy, Precision, Recall, F1 score, AUC-ROC, Log loss, Confusion Matrix are calculated to measure the performance of the global model. These metrics demonstrate model effectiveness in accurately classifying various types of kidney abnormalities. Each of the five chosen DL models employed in the framework undergoes the same federated learning and evaluation process. The best DL model is chosen based on the classification accuracy.

Proposed Federated Learning algorithm for Classification of Renal Disorders is expressed in Algorithm 1.

Algorithm 1 **Federated learning framework for kidney disorder classification.**


 1:  **Input**: CT kidney Dataset
 2:  **Output**: Best-performing DL model for classification in a federated environment
 3:  Load and prepare the CT kidney dataset
 4:  Resize images to 224 × 224 pixels
 5:  Normalize pixel values to [0, 1]
 6:  Split dataset into training and test sets (80:20)
 7:  Apply data augmentation (rotation, flip, brightness adjustment)
 8:  Initialize models: MobileNetV2, EfficientNetV2-S, ResNet50, DenseNet121, InceptionResNetV2
 9:  Distribute training data among *N* clients using stratified K-fold
10:  Build global DL model using the selected architecture
11:  **for** each global training round 1 to *P* **do**
12:     Randomly choose three clients from the seven participating clients
13:     **for** each selected client **do**
14:        Initialize global model weights
15:        Perform local training for *E* epochs
16:        Encrypt and send updated weights to server using AES
17:     **end** **for**
18:     **Server-side operations:**
19:     Decrypt all received weights
20:     Aggregate weights using FedWAvg
21:     Update global model parameters
22:  **end** **for**
23:  Assess the performance of the final global model on a centralized test dataset
24:  Compare performance of MobileNetV2, EfficientNetV2-S, ResNet50, DenseNet121, InceptionResNetV2
25:  Based on evaluation metrics, choose the best-performing model

To ensure reproducibility and provide technical clarity, Table 2 summarizes the key hyperparameters and configuration settings adopted in the proposed FL frame work.

**Table 2:** Hyperparameter configuration of the proposed federated learning framework.

Hyperparameter	Value
Image input size	224 × 224
Train : test split	80 : 20 (Stratified)
Number of clients	7
Clients selected per round	3 of 7
Global FL rounds	10
Client local epochs	5
Batch size	10
Learning rate	1 × 10^−4^
Optimizer	Adam
Loss function	Log loss
Activation (Output layer)	Softmax
Encryption	AES-256
Aggregation strategy	FedWAvg

### 3.5 AES encryption and decryption

In the proposed federated learning framework, a symmetric encryption scheme using the AES-based Fernet algorithm is employed to secure model weight transmissions. During each communication round, the server randomly selects three of the seven participating clients for local training. Each client receives the current global model, trains locally on its dataset, and subsequently encrypts its updated model weights using the shared symmetric key *k*. The encryption process is shown in Equation 5.


$$\begin{array}{l} c_{j}={\text{Enc}}_{k}\left(w_{j}\right) \end{array}$$ (5)

where *w*_*j*_ represents the local weights of client *j*, and *c*_*j*_ represents the resulting ciphertext. To preserve the confidentiality of model updates during transmission, encryption is performed immediately after local training on the client side. This step ensures that the encrypted model parameters are transmitted over the network and protects the sensitive information. Similarly, before the aggregation step, the decryption is carried out by the server. This preserves the privacy of individual client updates, and reduces the risk of sensitive information leakage.

From the server end, the ciphertext can be decrypted using the same symmetric key as shown in Equation 6.


$$\begin{array}{l} {ŵ}_{j}={\text{Dec}}_{k}\left(c_{j}\right) \end{array}$$ (6)

where ŵ_*j*_ represents the recovered model weights of client *j* after decryption. To update the global model, the decrypted weights from all the clients are aggregated using FedWAvg algorithm. The Fernet module ensures both confidentiality and integrity by using AES encryption in CBC mode with a 128-bit key, along with HMAC for message authentication. AES operates on 128-bit blocks and employs a key expansion method to generate a series of round keys from the original cipher key. This key schedule is expressed in Equation 7 (Daemen and Rijmen, 2002).


$$\begin{array}{l} E\left[j\right]=\{\begin{array}{lll} K\left[j\right], & \text{for }j<N\\ E\left[j-N\right]⊕\text{SubWord} & \text{if }j\;\mathrm{mod}\;N=0\\ \left(\text{RotWord}\left(E\left[j-1\right]\right)\right)⊕\text{Rcon}\left[j/N\right],\\ E\left[j-N\right]⊕\text{SubWord}\left(E\left[j-1\right]\right), & \text{if AES-256 and } & j\;\mathrm{mod}\;N=4\\ E\left[j-N\right]⊕E\left[j-1\right], & \text{otherwise} \end{array} \end{array}$$ (7)

where *E*[*j*] is the *j*-th word of the expanded key, *K*[*j*] is the *j*-th word of the original cipher key, and *N* is the key length in 32-bit words.

RotWord(*E*[*j* − 1]) performs a cyclic left byte rotation on the word *E*[*j* − 1].

SubWord applies AES S-box substitution to each byte of the word.

Rcon[*j*/*N*] denotes a round constant that is unique for each round. After completion of local training, each client encrypts its updated model parameters using AES-256 in Cipher Block Chaining (CBC mode) before sending them to the central server. Upon receiving the encrypted parameters, the server decrypts them and performs the FedWAvg aggregation to generate the updated global model. The updated global model is then distributed to the participating clients for the next communication round. This procedure is repeated throughout all the communication rounds (10) in the proposed framework. By encrypting model parameters during transmission, the framework ensures that model parameters transmitted in plaintext and provides protection against unauthorized access to model parameters during transmission. Integrating the encryption technique with the federated learning framework ensures a robust balance between model performance and communication privacy, enabling secure collaboration among clients without exposing raw model updates, thereby aligning with privacy-preserving goals in federated learning.

### 3.6 Federated weighted averaging (FedWAvg)

The key factor underlying the success of FL is the aggregation process. Federated Averaging (FedAvg), client model updates are aggregated using weights based only on the size of the local training dataset, without considering the performance of the locally trained models (Moshawrab et al., 2023). However, the limitation of FedAvg is, clients with poorly trained models but large datasets may disproportionately influence the global model update, resulting in degrading overall model performance. Inspired by performance-based aggregation strategies that incorporate local training accuracy into client weighting during aggregation (Huang et al., 2022), we introduce FedWAvg, which addresses this limitation by considering both the local dataset size and the local validation accuracy during the aggregation process. This method is particularly suitable for the proposed federated learning setup, where client datasets are generated using stratified partitioning to maintain similar class distributions across clients. The local model performance may still vary across clients due to variations in data characteristics within each partition. By considering local validation accuracy during aggregation, FedWavg assigns greater aggregation weight to more reliable client updates. In addition, this approach is better aligned with real-world clinical FL environments, where both data volume and model performance may differ across institutions.

Algorithm 2 represents the steps involved in the FedWAvg, which starts with the initialization of the global model weights *w*_0_. In every communication round *r*, three clients are randomly selected from the seven participating clients, denoted by *C*^*r*^, is selected using stratified sampling to ensure a more balanced representation of the client population. Each selected client k independently trains its local model on its dataset, updates the model weights *w*_*k*_, and computes the local data size *n*_*k*_, and evaluates its local validation accuracy *acc*_*k*_. This decentralized training enables each client to contribute to the global model based on its local data distribution, thereby enhancing the generalizability of the aggregated global model. After local training, each client transmits its updated model weights *w*_*k*_, local dataset size *n*_*k*_, and local validation accuracy *acc*_*k*_ to the central server.

Algorithm 2 Federated weighted averaging (FedWAvg). 
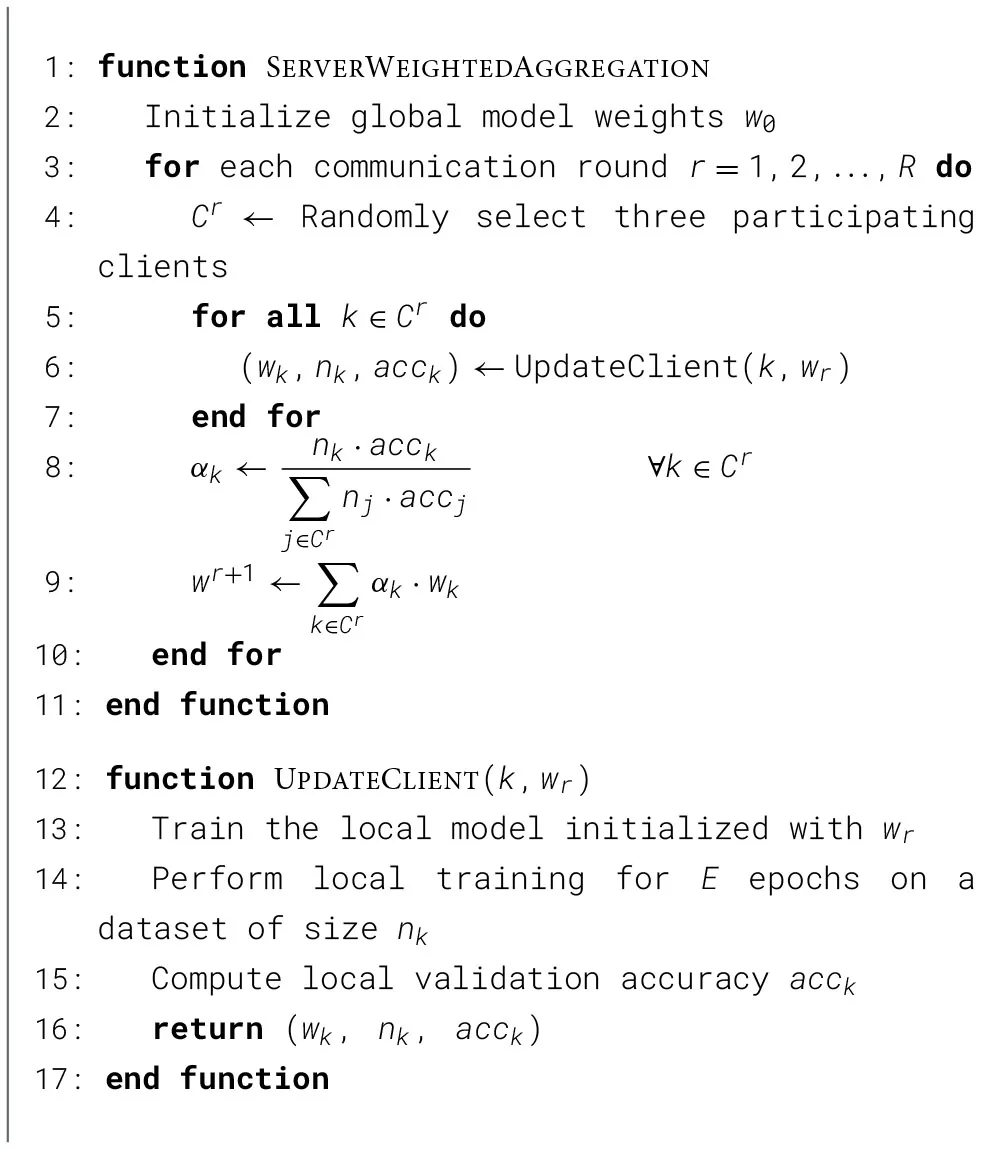

The server then computes a weighted average of the received local client model weights, incorporating both dataset size and local model accuracy, to update the global model weights, as expressed in given Equation 8


$$\begin{array}{l} w^{\left(r+1\right)}=\underset{k\in C^{r}}{\sum }\left(\frac{n_{k}\cdot acc_{k}}{\sum_{j\in C^{r}}n_{j}\cdot acc_{j}}\cdot w_{k}\right) \end{array}$$ (8)

Where:

- *w*^(*r*+1)^ represents the updated global model weights after the communication round (*r* + 1),
- *C*^*r*^ represents the set of clients in round *r*,
- *k* represents an individual client in the selected client set *C*^*r*^,
- *w*_*k*_ is the local model weights trained by client *k*,
- *n*_*k*_ is the local data samples on client *k*,
- *acc*_*k*_ is the local training accuracy of client *k*,
- $\underset{j\in C^{r}}{\sum }n_{j}\cdot acc_{j}$ is the normalizing factor ensuring all client weights sum to 1.

The UpdateClient function executes the local training process. Each of the clients initializes its model using the current global weights received from the server and trains locally for *E* epochs. After training, the client returns both the updated model weights *w*_*k*_ and local data size *n*_*k*_, and local validation accuracy *acc*_*k*_ to the server. The whole process, including client selection, local training, and weighted aggregation, continues iteratively for a fixed number of communication rounds or until model convergence.

### 3.7 Pre-trained models for federated learning

In the proposed FL framework, five benchmark Transfer Learning models are employed to strengthen feature representation and also reduce computational complexity and training time. In medical image analysis, these pre-trained models are widely recognized for effective knowledge transfer and reducing the need for large-scale labeled data, allowing the model to adapt efficiently with limited annotations and then improve generalization and accelerate convergence in an FL environment.

**MobileNetV2**—A lightweight convolutional neural network architecture, developed by Google, optimized for efficient performance on mobile and embedded devices. Its uniqueness of this variant lies in the incorporation of inverted residual blocks and linear bottlenecks which help maximize accuracy while minimizing computational cost (Sandler et al., 2018):

The operation of the inverted residual block can be expressed as Equation 9:


$$\begin{array}{l} z^{\prime }=z+F_{\text{inv}}\left(z\right) \end{array}$$ (9)

Where:

- *z* denotes the input feature map to inverted residual block.
- *F*_inv_(*z*) denotes the inverted residual transformation, which includes an expansion using 1 × 1 convolution, depthwise separable convolution, and a linear bottleneck projection.
- *z*′ represents the output feature map obtained after applying the skip (residual) connection.

By balancing speed, accuracy, fast inference, and low memory consumption, the MobileNetV2 is particularly well-suited for medical imaging tasks. Depthwise separable convolutions greatly lower the number of parameters and computational overhead while maintaining performance. Its ability to capture fine-grained features makes it a valuable choice for medical image analysis (Indraswari et al., 2022).

**EfficientNetV2-S**—A computationally efficient convolutional neural network model from the EfficientNetV2 family, designed to optimize model performance by carefully balancing the size and complexity of the network in a structured and optimized manner. As a smaller and faster variant, and also balances between accuracy and computational efficiency, EfficientNetV2-S is highly suitable for medical imaging tasks such as detection and classification. Its architecture is composed of multiple MBConv (Mobile Inverted Residual Bottleneck) blocks, which are specifically engineered to learn complex features efficiently and to reduce computational complexity while keeping the model lightweight and fast (Abd El-Aziz et al., 2024). The Mathematical operation involved in MBConv block can be expressed as Equation 10 (Tan and Le, 2021).


$$\begin{array}{l} b=a+F_{MBConv}\left(a\right) \end{array}$$ (10)

Where:

- *a* represents the input feature map to the MBConv block.
- *F*_*MBConv*_(*a*) denotes the non-linear transformation applied to *x*, comprising of 1 × 1 convolution, depthwise 3 × 3 convolution, projection 1 × 1 convolution back to a lower dimension and an optional squeeze and excitation module.
- *b* denotes the output feature map after the residual connection.

**ResNet50**—Is a 50-layer deep CNN developed by Microsoft to overcome the vanishing gradient problem in deep architectures. It utilizes residual connections, which facilitate gradient flow more effectively during back propagation, ensuring stable and efficient learning. The computational operations of bottleneck residual block can be represented in Equation 11 (He et al., 2016), ensuring stable and efficient learning.


$$\begin{array}{l} b=a+F_{\text{ResNet}}\left(a\right) \end{array}$$ (11)

Where:

- *a*: input feature map to the bottleneck block.
- *F*_ResNet_(*a*): bottleneck transformation applied to *a*, compraising of 1 × 1 convolution for channel reduction, followed by a 3 × 3 convolution for spatial feature extraction, and a final 1 × 1 convolution to restore the original channel dimension.
- *b* : output feature map after the residual (skip) connection.

Maintaining high accuracy, robust feature extraction, and efficient training, ResNet50 is particularly suitable for medical image tasks, where capturing the fine structural details is essential. Pre-trained ResNet50 on Imagenet enables the model to learn general features from a wide range of images, which can be fine-tuned for specific tasks such as disease detection, classification in medical images (Hossain et al., 2022).

**DenseNet121**–Is a deep convolutional network developed to improve feature propagation and handle the vanishing gradient problem, in which each layer receives inputs from all previous layers, which helps the model learn more efficiently. DenseNet121 is a variant from the DenseNet family with 121 layers. The operation of a dense layer can be expressed in Equation 12 (Huang et al., 2017).


$$\begin{array}{l} y_{k}=H_{k}\left(\left[y_{0},y_{1},\ldots ,y_{k-1}\right]\right) \end{array}$$ (12)

Where:

- *y*_0_, *y*_1_, …, *y*_*k*−1_ represent the feature maps generated by all preceding layers.
- [·] indicates the concatenation of these feature maps along the channel dimension.
- *H*_*k*_(·) refers to the transformation function at layer *k*, including Batch Normalization (BN), ReLU activation, a 1 × 1 bottleneck convolution, and a 3 × 3 convolution.
- *y*_*k*_ is the output feature map produced by the *k*^th^ layer.

The transition layers and bottleneck helps to maximize the performance and to minimize the computational complexity. DenseNet121 provides significant advantages in terms of effective parameter utilization, higher accuracy, and faster convergence, making it particularly effective for medical imaging applications that require detailed feature extraction. It enhances diagnostic precision through fine-grained visual patterns capturing ability and operates effectively with minimum training data (Arulananth et al., 2024).

**InceptionResNetV2**—Is a deep convolutional network that incorporates Inception modules with residual connections, allowing it to efficiently capture hierarchical features while maintaining stable and efficient gradient propagation during training. This hybrid architecture mitigates the vanishing gradient problem, while improving the convergence speed and accuracy. Its architecture is composed of three main units, including the Stem, multiple Inception Resnet-A/B/C blocks, and a Reduction block in between each type, which facilitates the model to progressively learn more abstract representations as the depth of the network increases. The functioning involved in residual inception block is expressed in Equation 13 (Szegedy et al., 2017):


$$\begin{array}{l} b=a+\alpha F_{\text{IR}}\left(a\right) \end{array}$$ (13)

Where:

*a*: input feature map to Inception-ResNet block.

*F*_IR_(*a*): inception-style transformation applied to *a*, comprising various parallel convolutional branches whose outputs are then concatenated and merged using a 1 × 1 convolution.

α: residual scaling factor (typically α = 0.1) used to stabilize training in deep residual networks.

*b*: output feature map after applying the scaled residual connection.

The models' ability to capture fine-grained and hierarchical features is significantly advantageous for identifying subtle abnormalities. The modular architecture of InceptionResnetV2 makes it highly effective for medical image analysis, particularly in the classification of anomalies (Sunsuhi and Jose, 2022).

## 4 Results and discussion

This section outlines the results obtained from the proposed federated learning framework, starting with hardware and software environment and training configuration, followed by the performance metrics used, experimental results, and the strengths and limitations of its application in real-world clinical settings.

### 4.1 Implementation platform

The proposed framework was implemented on Kaggle cloud computing platform using Python 3.10 (Python Software Foundation, Beaverton, OR, USA), with experiments conducted on a Tesla T4 GPU with 15 GB of GPU memory and approximately 13 GB of RAM. The DL models were developed using PyTorch 2.0 (PyTorch Foundation, USA) with the torchvision library, with IncetionResNetV2 loaded with timm library. All images were processed using OpenCV, resized to 224 × 224 pixels, and normalized using ImageNet mean and standard deviation values. AES-256 encryption in CBC mode was implemented using the PyCryptodome library to secure model parameter transmission between clients and the central server, and model performance was evaluated using the scikit-learn library. Some result plots were generated using OriginPro for clearer visual presentation.

The proposed FL framework involved seven participating clients, each client trained independently using five pre-trained deep learning models for the classification of renal disorders. Federated weighted averaging was employed to aggregate client updates, and AES-256 encryption was integrated to ensure the secure transmission of model parameters. The training process applied Adam optimizer with a learning rate of 0.0001, a batch size of 10, five local client epochs and 10 global rounds.

### 4.2 Evaluation metrics

The proposed model is assessed using different performance metrics.

Accuracy—Evaluates the ratio of correct predictions to the total number of samples, as expressed in Equation 14.


$$\begin{array}{l} \text{Accuracy}=\frac{T_{P}+T_{N}}{T_{P}+F_{P}+F_{N}+T_{N}} \end{array}$$ (14)

Recall—Quantifies the model's effectiveness in identifying true positive instances from all actual positive samples. The recall is calculated using Equation 15.


$$\begin{array}{l} \text{Recall}=\frac{T_{P}}{F_{N}+T_{P}} \end{array}$$ (15)

Precision—Represents the proportion of correctly identified positive samples to the total number of samples predicted as positive, represented in Equation 16.


$$\begin{array}{l} \text{Precision}=\frac{T_{P}}{T_{P}+F_{P}} \end{array}$$ (16)

F1 Score—Measures the balance between a model's precision and recall in a single metric, as expressed in Equation 17.


$$\begin{array}{l} \text{F1 Score}=\frac{2\times \left(\text{Precision}\times \text{Recall}\right)}{\text{Precision}+\text{Recall}} \end{array}$$ (17)

Log Loss—Measures the uncertainty of the predicted class probabilities with respect to the true labels, as represented in Equation 18.


$$\begin{array}{l} \text{Log Loss}=-\frac{1}{N}\left(\overset{N}{\underset{i=1}{\sum }}\overset{C}{\underset{j=1}{\sum }}y_{ij}\log\left({ŷ}_{ij}\right)\right) \end{array}$$ (18)

Where:

- *N* represents the total number of samples.
- *C* represents the number of classes.
- *y*_*ij*_ is the true label indicator for sample *i* and class *j*.
- ŷ_*ij*_ is the predicted probability of sample *i* belonging to class *j*.

Area under the Receiver Operating Characteristic Curve (AUC-ROC)—This metric is utilized to assess the ability of a classification model to distinguish between classes. For each class, it treats that class as “positive” and the others as “negative.” The overall score is calculated by averaging the AUC-ROC of all the classes, as shown in Equation 19.


$$\begin{array}{l} {\text{AUC}}_{n}=\int_{0}^{1}{\text{TPR}}_{n}\left({\text{FPR}}_{n}\right)d\left({\text{FPR}}_{n}\right) \end{array}$$ (19)

Final macro-AUC is shown in Equation 20.


$$\begin{array}{l} \text{Macro}=\frac{1}{4}\overset{4}{\underset{n=1}{\sum }}{\text{AUC}}_{n} \end{array}$$ (20)

### 4.3 Performance evaluation

This section presents the performance evaluation of the five pre-trained models trained under the proposed FL framework. Each model was evaluated on accuracy, loss, precision, recall, F1 score, AUC-ROC after 10 global rounds. Confusion matrices are also presented to provide a detailed view of the classification performance across all models.

**Figure 2:**
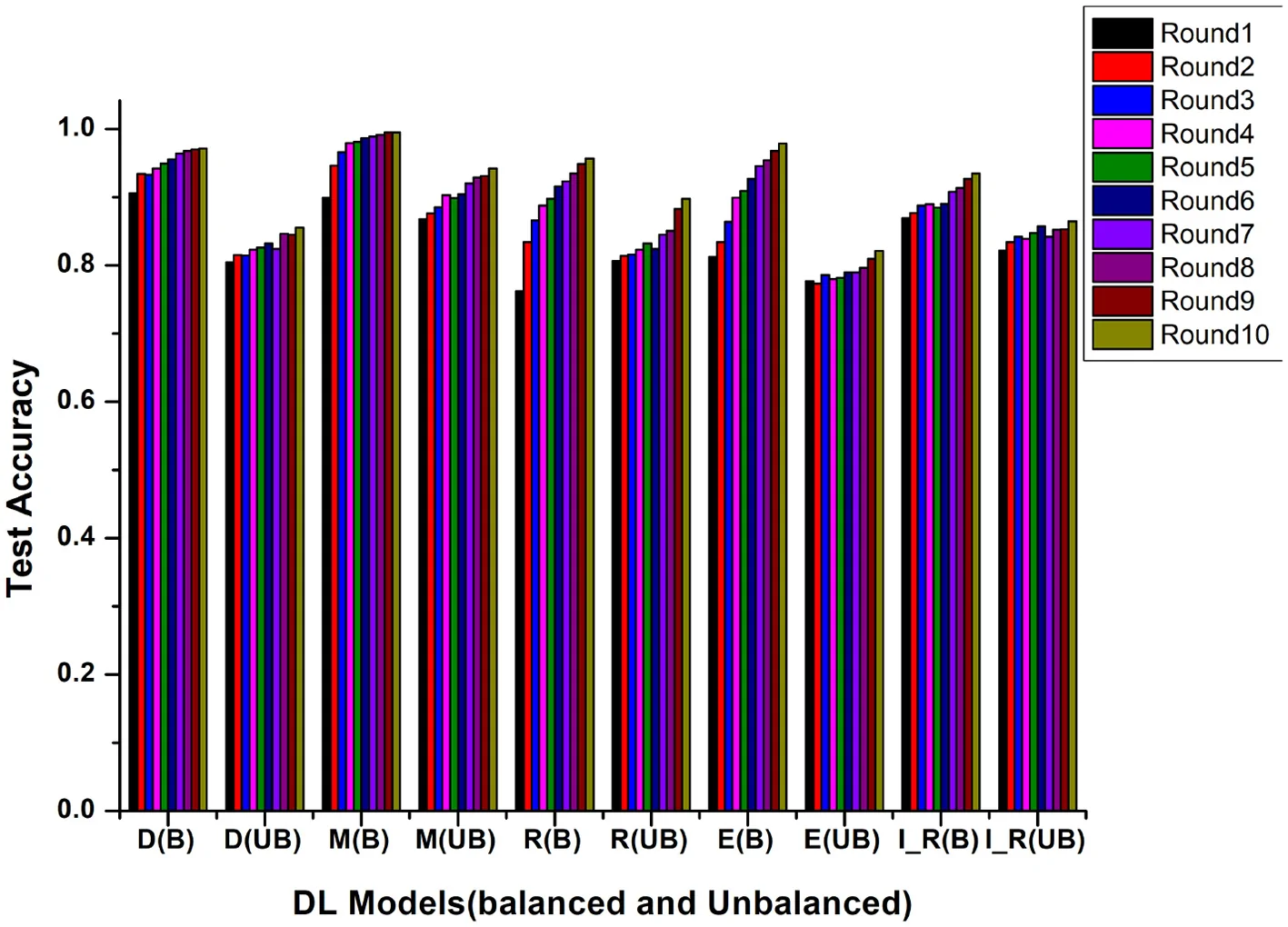
Comparison of test accuracy on balanced and imbalanced datasets across DL models.

The graph (Figure 2) shows a comparative analysis of multiple pre-trained DL models in a federated learning (FL) environment, employed on both balanced (B) and unbalanced (UB) datasets. In the graph, D, M, R, E, and I represent DenseNet121, MobileNetV2, ResNet50, EfficientNetV2, and Inception-ResNetV2, respectively. When trained with balanced datasets, all models achieve higher accuracy compared to their unbalanced counterparts ranging from 5.27 to 15.72%. At the 10th round, EfficientNetV2 exhibits the highest gain (15.72%), followed by DenseNet121 (11.57%). ResNet50 and Inception-ResNetV2 show moderate improvement (7.01 and 5.91%, respectively). MobileNetV2 exhibited the lowest improvement (5.27%). Overall, the results clearly indicate that balanced datasets lead to better classification accuracy across all DL models in the FL environment.

**Figure 3:**
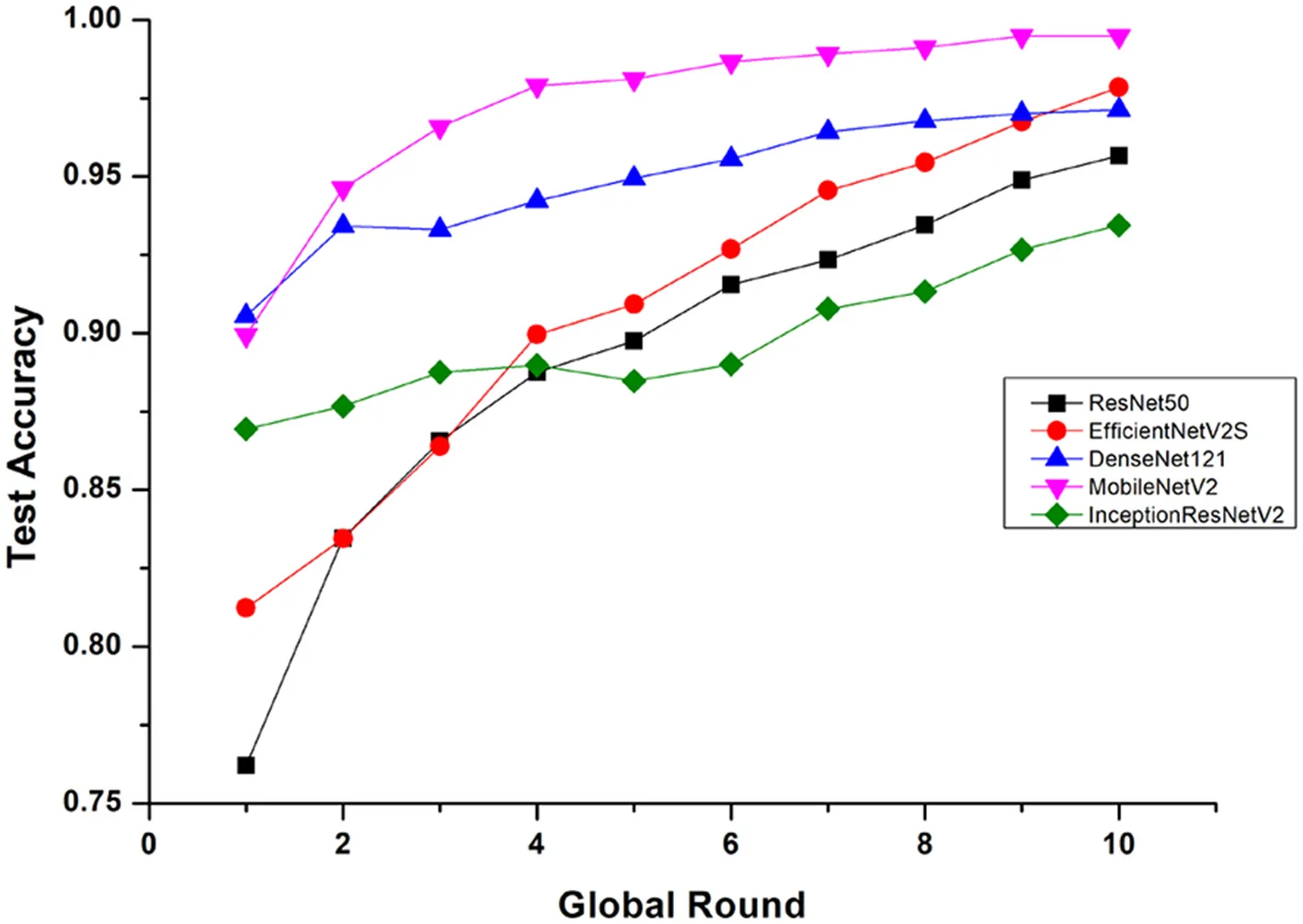
Test accuracy across global rounds for different pre-trained DL models.

The graph (Figure 3) shows the test accuracy obtained across 10 epochs for the five DL models in the FL environment. The results demonstrate consistent improvement across rounds. In round 1, ResNet50 achieved 76.21%, EfficientNetV2-S reached 81.23%, DenseNet121 recorded 90.56%, MobileNetV2 achieved 89.94%, and InceptionResNetV2 attained 86.95%. By epoch 10, MobileNetV2 achieved the highest test accuracy of 99.48%, followed by EfficientNetV2-S with 97.79%, and DenseNet121 with 97.15%. ResNet50 with 95.66 %, while InceptionResNetV2 attains 93.49% accuracy. Despite minor fluctuations were obsedved in certain rounds, the overall progression demonstrates stable convergence and effective learning within the FL framework.

**Figure 4:**
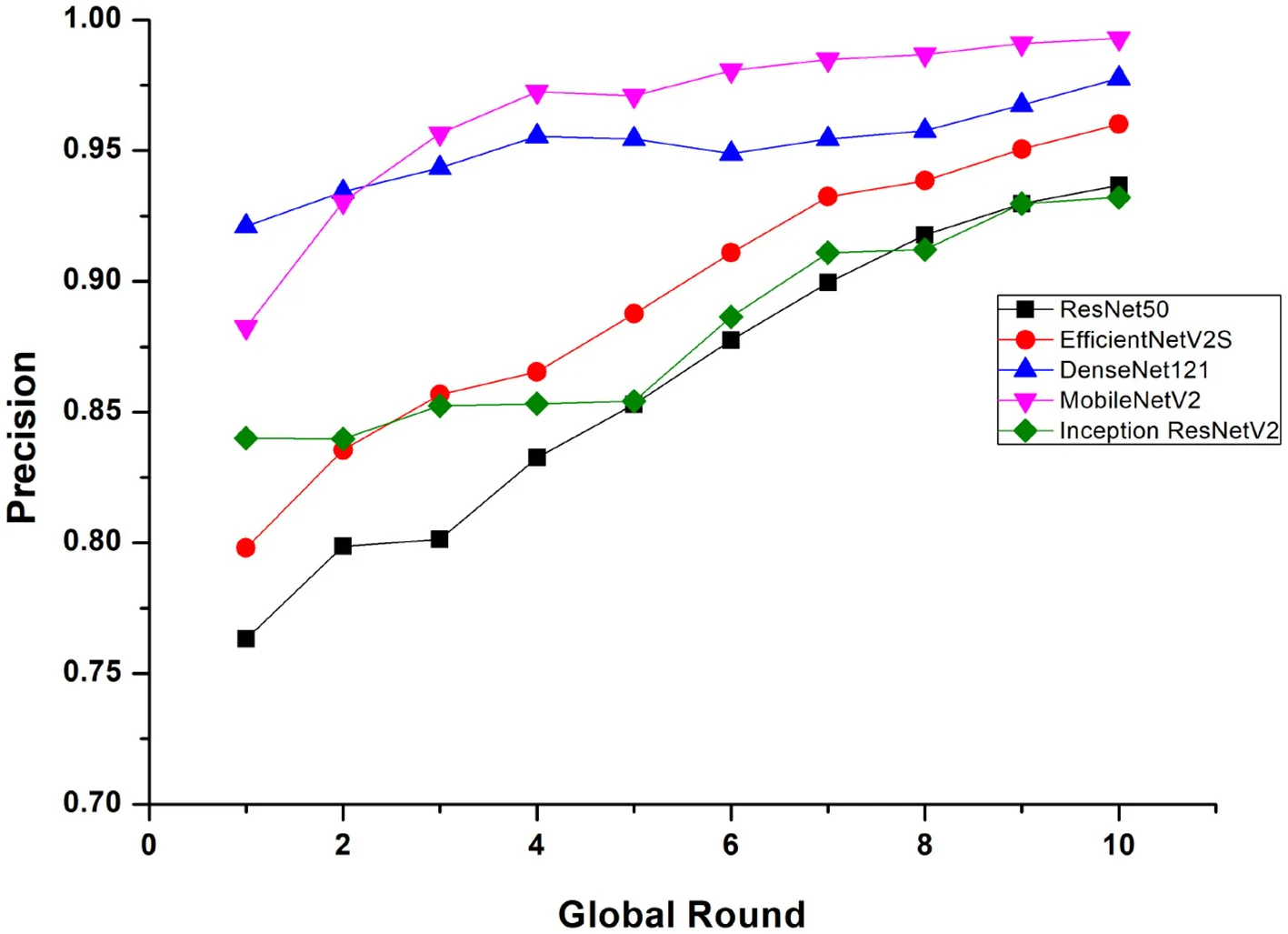
Test precision across global rounds for different pre-trained DL models.

The graph (Figure 4) illustrates the precision observed for the five DL models, demonstrating a consistent improvement in the models' capabilities within the FL framework. The precision values progressively increase across most of the models, with MobileNetV2 achieving the highest precision of 99.29%, followed by DenseNet121 with 97.77%, EfficientNetV2-S with 96.01%, ResNet50 with 93.67%, and InceptionResNetV2 with 93.21% at global epoch 10. This upward trend indicates a lower false positive rate and improved precision in positive class identification.

**Figure 5:**
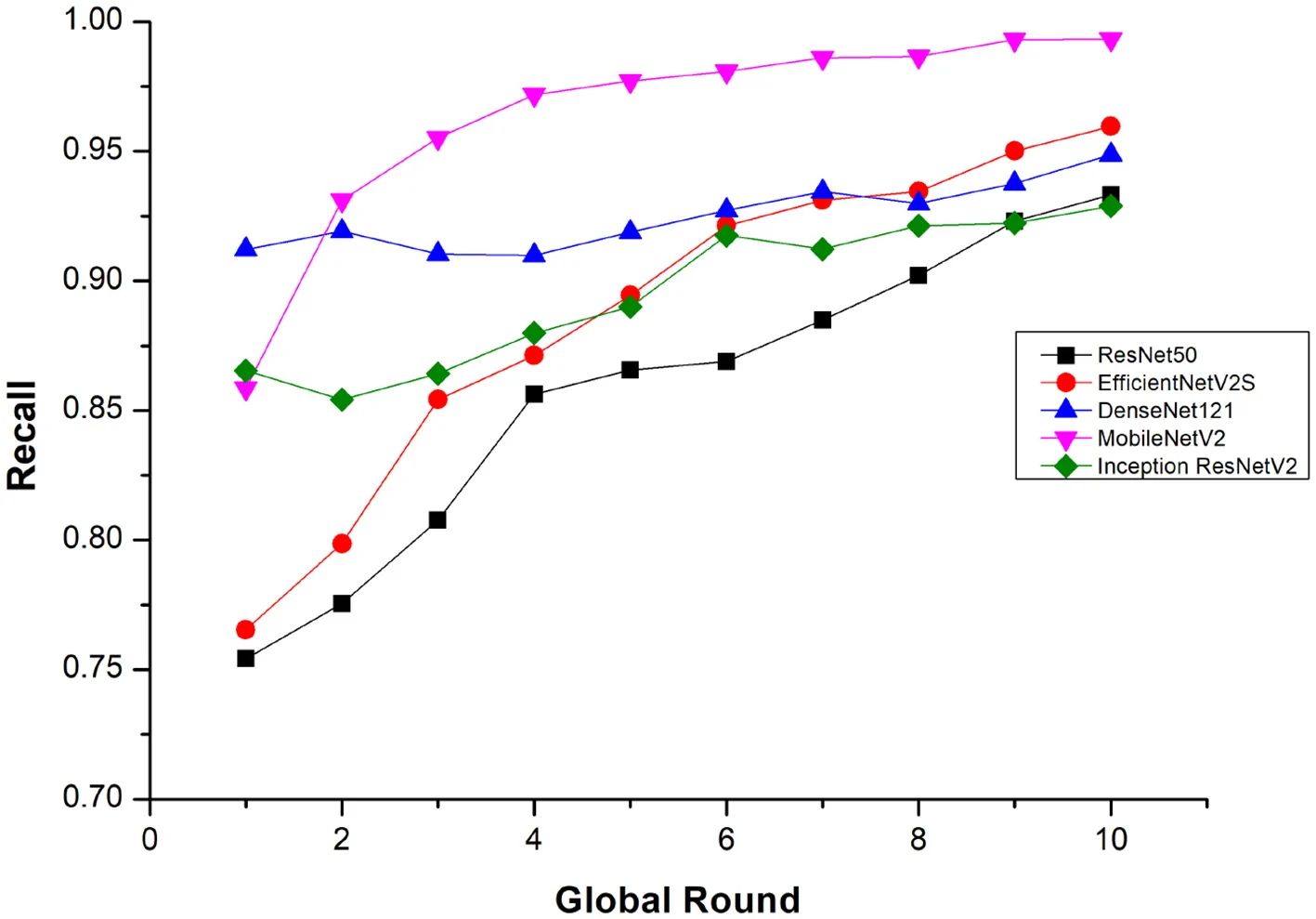
Test recall across global rounds for different pre-trained DL models.

Regarding Recall, represented by Figure 5, the recall values at epoch 10 show that MobileNetV2 achieved the highest recall of 99.32%, followed by EfficientV2S with 95.96%, DenseNet121 with 94.87%, InceptionResNetV2 with 92.89%, and ResNet50 with 93.32% Although minor fluctuations are present in the earlier rounds, the progressive uptrend in recall highlights the models' capability to maintain reliable performance across different data distributions in a decentralized environment.

**Figure 6:**
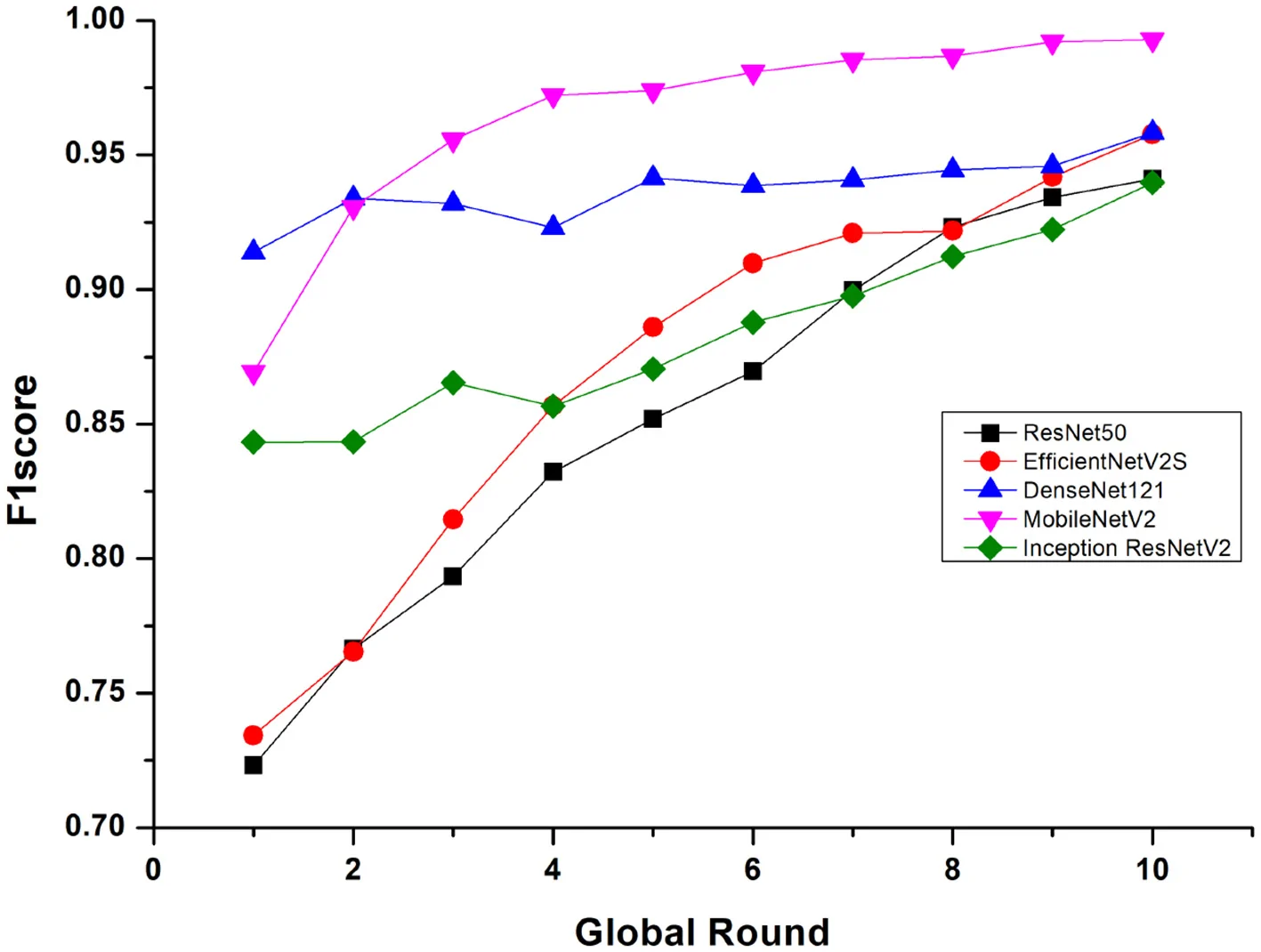
F1-score across global rounds for different pre-trained DL models.

The graph (Figure 6 shows the F1-score achieved across 10 global epochs for the five models in the FL environment, demonstrating improved model performance. By the 10th epoch, the scores rise steadily, with MobileNet achieving the highest F1-score of 0.993, followed by DenseNet121 with 0.9584, EfficientNetV2-S with 0.9578, ResNet50 with 0.9412, and InceptionResNetV2 with 0.9398.

**Figure 7:**
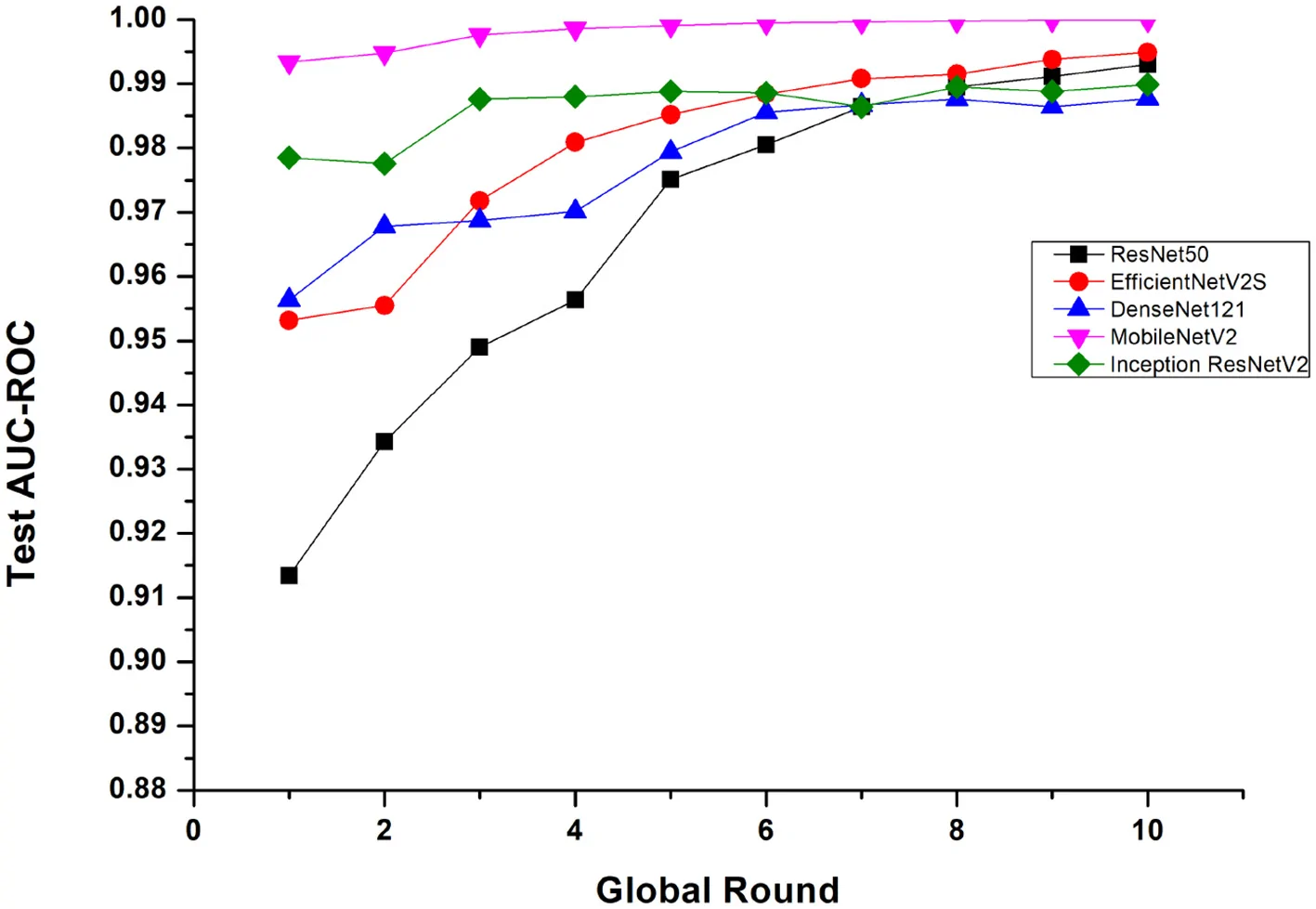
AUC-ROC across global rounds for different pre-trained DL models.

The graph (Figure 7) shows the AUC values exhibit strong classification capability across epochs, the AUC values recorded at the 10th epoch vary across models, with ResNet (0.993), EfficientNetV2-S (0.9949), DenseNet121 (0.9877), InceptionResNet (0.9899), and MobileNet (0.9999). This consistent upward trend demonstrates balanced performance and also sustained ability to distinguish between different classes under distributed and heterogeneous conditions. Overall, the results show that MobileNetV2 gives the unsurpassed classification capability in terms of AUC, as compared with EfficientNetV2-S, ResNet, DenseNet121 and InceptionResNetV2.

**Figure 8:**
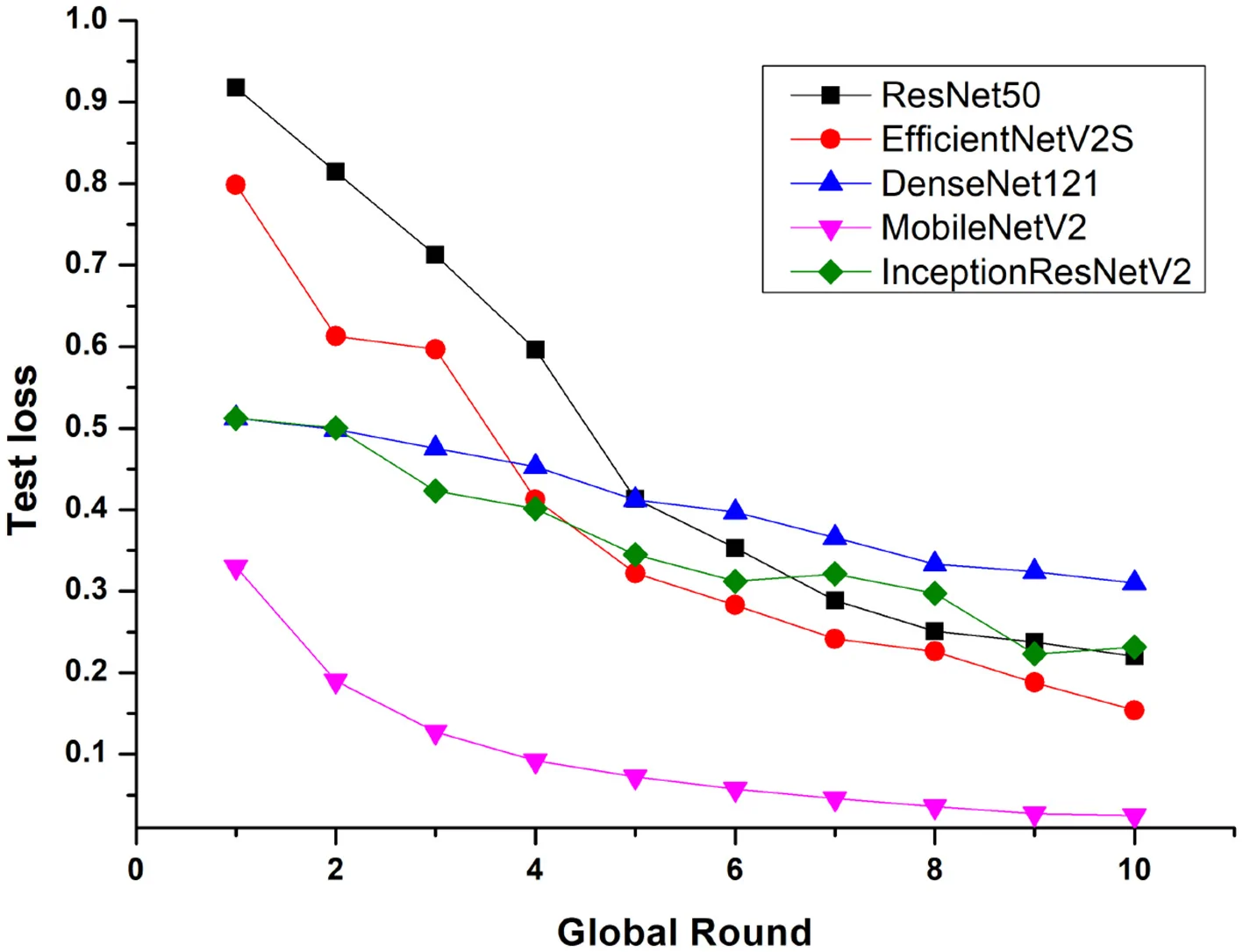
Test loss across global rounds for different pre-trained DL models.

The graph (Figure 8) shows the loss values obtained across 10 global rounds in the federated learning setup for five DL models. This shows a consistent downward trend, reflecting effective model optimisation and convergence. During round 1, all models exhibit relatively high loss values ranging from 0.3300 to 0.9176. Although there are some minor fluctuations across the rounds, the consistent decline across multiple rounds highlights the ability of the FL framework to minimize error, enhance learning stability, and adapt efficiently on heterogeneous and distributed client data. By round 10, MobileNetV2 achieves the lowest loss value of 0.0247, followed by EfficientNetV2-S with 0.1540, DenseNet with 0.3102, InceptionResNetV2 with 0.2313, and ResNet50 with 0.2204.

Table 3 presents the performance comparison of five pre-trained DL models after the 10th Global round of federated training.

**Table 3:** Performance comparison of pre-trained deep learning models after the 10th global round of federated training.

Model	Accuracy	Loss	Precision	Recall	F1-Score	AUC-ROC
MobileNetV2	0.9948	0.0247	0.9929	0.9932	0.9930	0.9999
EfficientNetV2-S	0.9779	0.1540	0.9601	0.9596	0.9578	0.9949
DenseNet121	0.9715	0.3102	0.9777	0.9487	0.9584	0.9877
ResNet50	0.9566	0.2204	0.9367	0.9332	0.9412	0.9930
InceptionResNetV2	0.9349	0.2313	0.9321	0.9289	0.9398	0.9899

Table 3 shows that all pre-trained models achieved high classification performance after the 10th global round of federated training. MobileNetV2 obtained the highest accuracy (99.48%), followed by EfficientNetV2-S and DenseNet121, demonstrating the effectiveness of the proposed FL framework.

**Figure 9:**
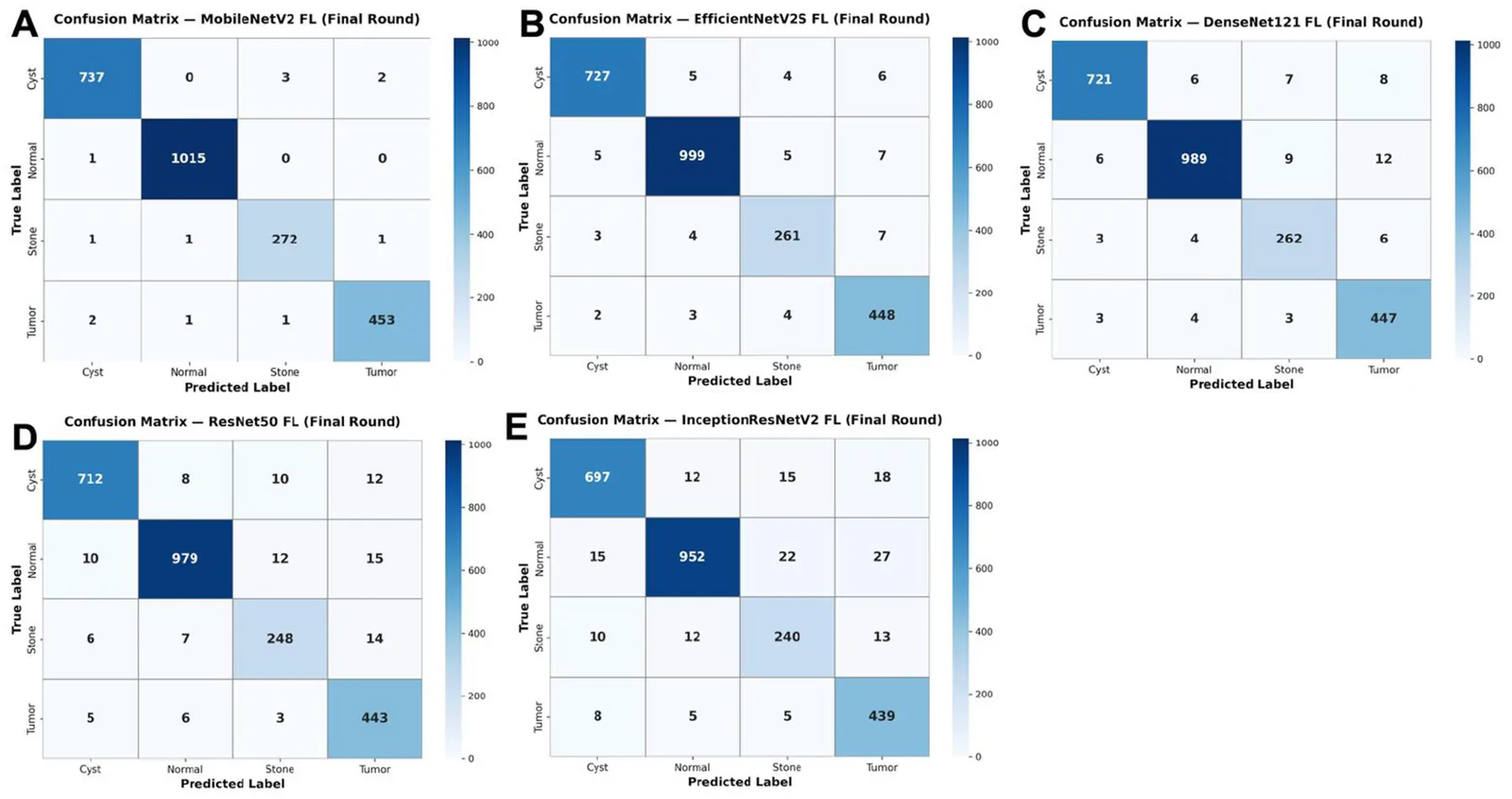
Confusion matrices for different pre-trained DL models: **(A)** MobileNetV2, **(B)** EfficientNetV2-S, **(C)** DenseNet121, **(D)** ResNet50, **(E)** InceptionResNetV2.

Figures 9A–E represents the confusion matrices for all five models, showing that MobileNetV2 achieved the highest per-class accuracy across all four kidney abnormalities, with 99.33% for Cyst, 99.90% for normal, 98.91% for stone, and 99.12% for Tumor, with an overall accuracy of 99.48%. The stone class exhibited the lowest classification accuracy across all models, possibly due to its similarity with other abnormalities. InceptionResNetV2 achieved the lowest accuracy of 93.49%, while the EfficientNetV2-S and DenseNet121 achieved comparable accuracies of 97.79 and 97.15,% respectively.

### 4.4 Computational efficiency

The computational efficiency of the five pre-trained models was evaluated in terms of training time, communication rounds, and communication overhead within the proposed FL framework. Training times were recorded with and without AES-256 encryption to assess its computational overhead.

Table 4 demonstrated that MobileNetV2 attained highest computational efficiency, completing training in 10.55 min with AES-256 encryption and 10.46 min without AES, corrsponding to an encryption overhead of only 0.86%, representing that secure parameter transmission incurs only a low computational cost. In contrast, InceptionResNetV2 required the longest training time of 79.80 min with the highest communication overhead of 623.57MB per round due to its larger model size. These results indicate that MobileNetV2 provides balance between classification performance and computational efficiency, making it a suitable architecture for practical deployement in federated clinical environments.

**Table 4:** Computational efficiency of five pre-trained models in the federated learning framework.

Model	Comm. rounds	Avg round time (s)	Train time w/ AES (min)	Train time w/o AES (min)	Comm. overhead per Round (MB)	Total Comm. overhead (MB)
MobileNetV2	10	63.28	10.55	10.46	26.21	262.02
ResNet50	10	169.41	28.23	27.58	270.03	2,700.27
EfficientNetV2-S	10	162.82	27.14	26.35	233.53	2,335.27
DenseNet121	10	152.86	25.48	24.62	81.35	813.47
InceptionResNetV2	10	478.82	79.80	73.78	623.57	6,235.71

### 4.5 Cross-client validation analysis

To address the possibility of overfitting, cross-client validation was performed by evaluating the final global model independently on each of the seven client partitions. The results are presented in Table 5.

**Table 5:** Client-wise data distribution and classification accuracy.

Client	Samples	Accuracy (%)
Client 0	2,321	99.01
Client 1	2,321	98.06
Client 2	2,321	98.58
Client 3	2,321	99.14
Client 4	2,320	99.31
Client 5	2,320	98.45
Client 6	2,320	99.40

Table 5 shows that the Global model maintained consistent performance across all clients, with accuracy values ranging from 98.06% (Client 1) to 99.40% (Client 6) and an average cross-client accuracy of 98.85%, and it was observed that the generalization gap between the cross-client accuracy and the test accuracy was −0.0063. These results indicate that the model's performance is due to its strong capability to generalize across different client datasets, suggesting good generalization and minimal overfitting.

### 4.6 Cross-domain generalizability study

Most of the prior works for kidney CT scan classification have been developed and validated using the same dataset, raises concerns regarding the reproducibility and generalizability of the reported outcomes. For the cross-domain generalizability assessment of the proposed methodology, a lung cancer dataset was chosen because of the strong clinical association between kidney and lung conditions. Patients with renal impairment are reported to have a higher risk of developing pulmonary malignancies (Shang et al., 2025; Sim et al., 2016). Since kidney function strongly impacts the progression and treatment of lung cancer, the Iraq-Oncology Teaching Hospital/National Center for Cancer Diseases (IQ-OTH/NCCD) lung cancer dataset publicly available in Kaggle, offers a relevant external validation for the proposed methodology https://www.kaggle.com/datasets/adityamahimkar/iqothnccd-lung-cancer-dataset. The dataset contains a total of 1,097 CT scan images from 110 patients categorized into malignant, normal, and benign.

To assess cross-domain generalizability, the proposed methodology was applied on lung cancer dataset using MobileNetV2 as it achieved the highest accuracy on the kidney CT scan dataset.

**Figure 10:**
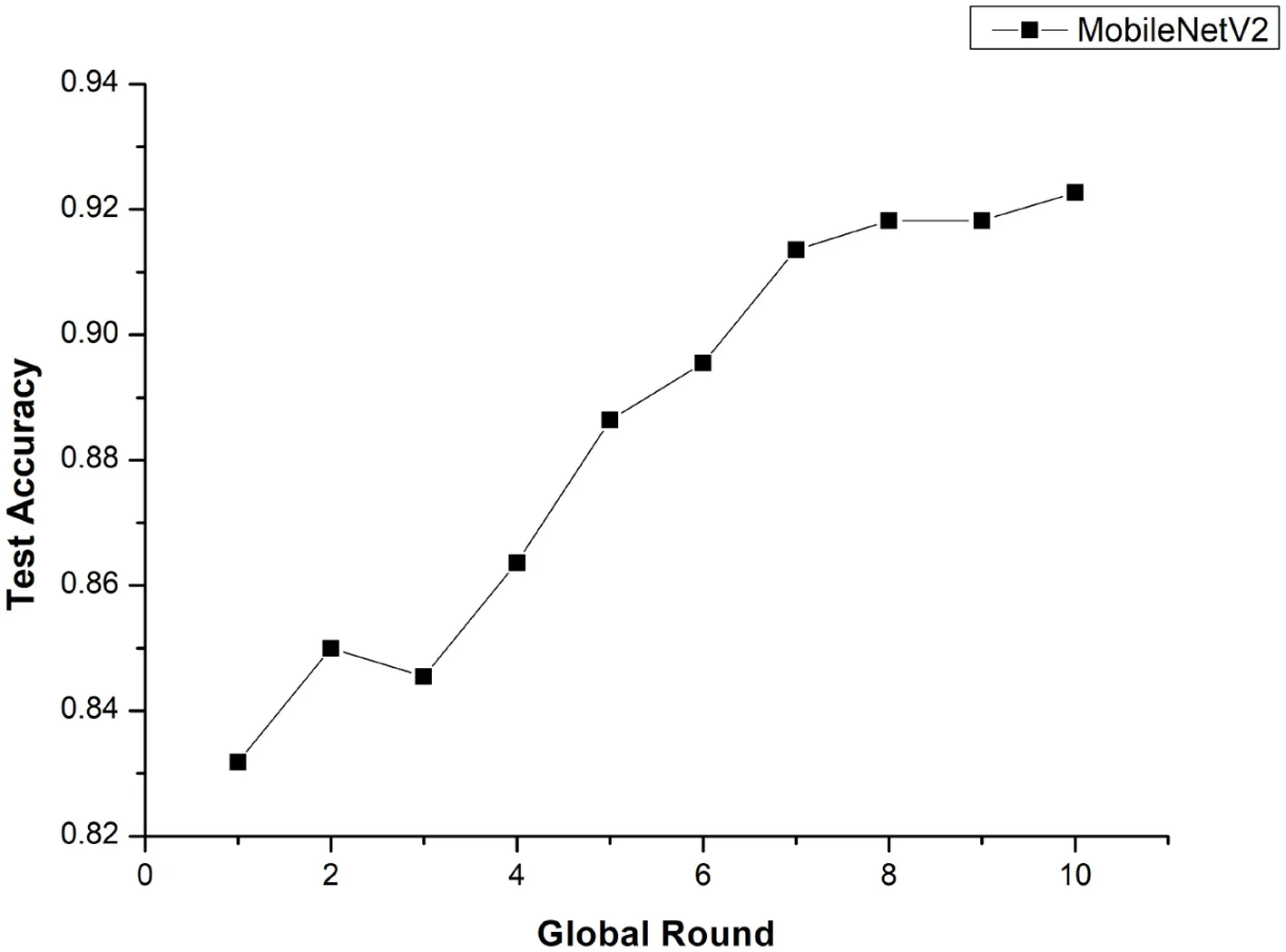
Test accuracy on lung cancer dataset.

The graph (Figure 10) shows that the test accuracy of MobileNetV2 improved steadily across global rounds, reaching about 92.27% by the 10th round after some initial fluctuations.

**Figure 11:**
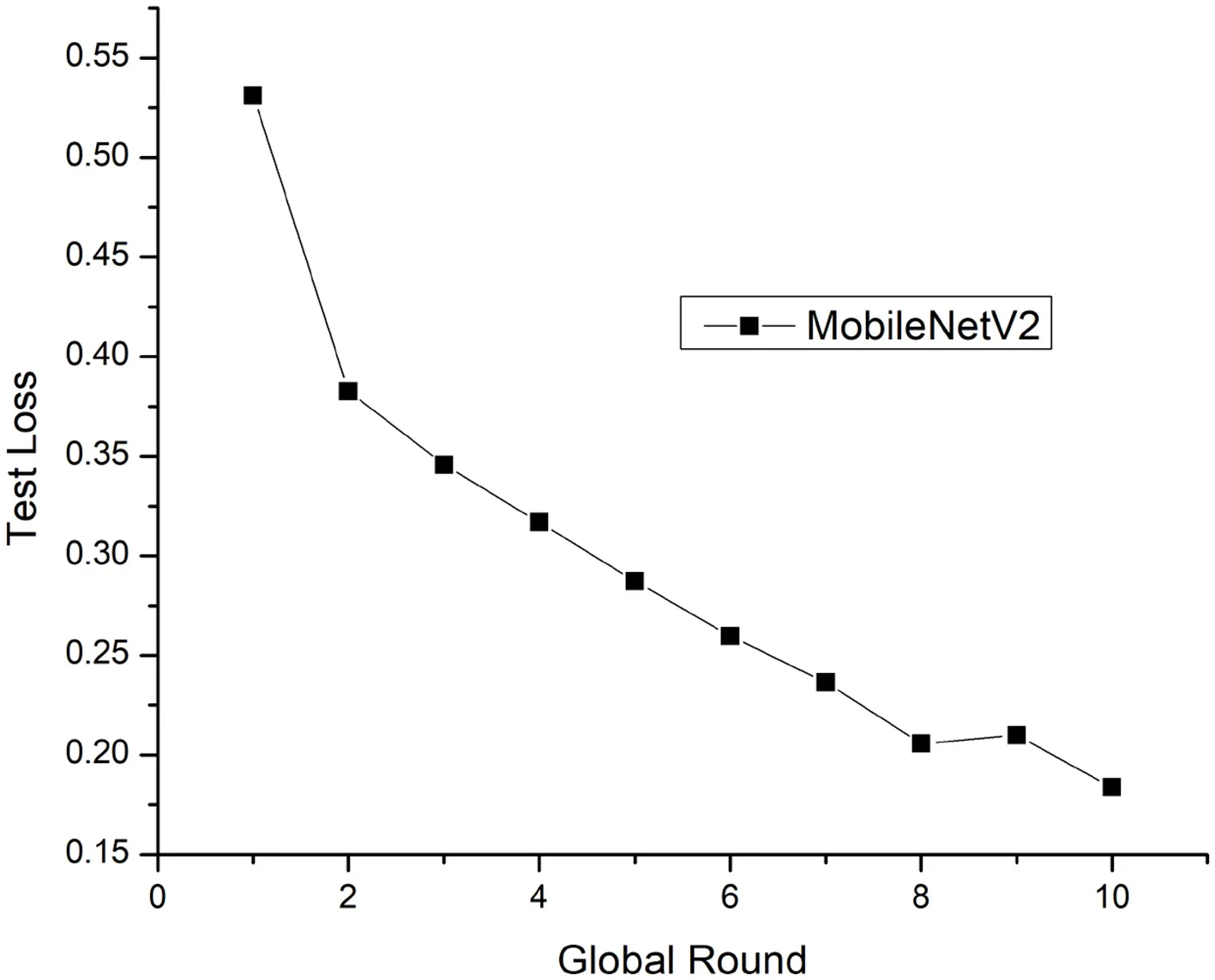
Test loss on lung cancer dataset.

The graph (Figure 11) demonstrates the test loss obtained using MobileNetV2 on the lung cancer dataset. The loss consistently declined during the initial rounds, ensuring effective learning. After Round 4, the downward trend continued steadily through Rounds 5–8. A minor fluctuation was observed between Rounds 8 and 9, where the loss slightly increased from 0.2057 to 0.2099. Overall, the decreasing loss trend confirms effective model optimization and convergence within the FL framework.

### 4.7 Selection of aggregation technique

In the federated learning Environment, four aggregation techniques, including FedWeighted Averaging, FedAveraging, FedProx, and FedAdam, are evaluated on the kidney CT scan dataset. FedWAvg technique aggregates client updates proportional to dataset sizes. FedProx extends the standard averaging by adding a proximal term, which helps stabilize training for handling heterogeneous client data. FedAdam improves standard Federated Averaging by applying adaptive optimization at the server-side aggregation for stable and faster convergence. In order to find out the efficient aggregation technique, we selected MobileNetV2 as a base model, as it provides relatively low execution time while maintaining reasonable accuracy. Using the Proximal Aggregation, MobileNetV2 achieved an accuracy improvement from 77% to 83%, Meanwhile, using FedAdam, the classifier achieved a comparatively lower range of 64% to 72% accuracy. FedAvg achieved an accuracy of 99.2% at the tenth global round, whereas FedWAvg provides more consistent progress over rounds and achieved an accuracy of 99.48% during the ninth global round.

### 4.8 Clinical applicability

In a real-world setting, each participating hospital or diagnostic center would act as a federated client, retaining its patient CT scan data locally. A central server would distribute the global model to selected institutions, where local training is performed on private datasets. The updated model parameters will be encrypted using AES-256 and securely transmitted to the server for aggregation using the FedWAvg algorithm. The aggregated global model would then be redistributed for subsequent training rounds. This collaborative process enables model improvement across institutions while preserving patient privacy. The low communication overhead of MobileNetV2 supports its practical deployment in healthcare environments with varying network capabilities.

## 5 Conclusion and future scope

The proposed model presents a secure federated learning framework for the classification of renal abnormalities, such as cyst, tumor, stone, and normal cases, using CT scan images from the Kaggle dataset. To handle class imbalance and to improve generalization, Data augmentation was performed on the training dataset to expand minority classes. Five pre-trained deep learning architectures such as ResNet50, MobileNetV2, EfficientNetV2-S, DenseNet121, and InceptionResNetV2 were incorporated within the FL framework to leverage transfer learning and enhance classification performance. The proposed framework involves multiple distributed clients performing local training using their assigned datasets and transmitting AES-encrypted model parameters to the central server, where the global model is progressively refined through a weighted aggregation of client updates. Experimental results demonstrate consistent performance improvement across global training rounds. Among the five models, MobileNetV2 delivered superior results, reaching a classification accuracy of 99.48% with per-class accuracies of 99.33% (Cyst), 99.90% (Normal),98.91% (Stone), and 99.12% (Tumor) and the lowest loss value of 0.0247, and AUC-ROC of 0.9999. Cross- client validation across all seven federated clients yielded an average accuracy of 98.85% with a generalization gap of −0.0063, confirming no 64% overfitting within the FL environment. EfficientNetV2-S and DenseNet121 also demonstrated strong performance with an accuracy of 97.79% and 97.15%, respectively. ResNet50 achieved competitive results with an accuracy of 95.66%. Meanwhile, InceptionResNetV2 achieved a comparatively lower accuracy of 93.49%.

The comparative analysis also revealed that balanced datasets significantly improve classification accuracy compared to unbalanced datasets in the federated learning environment. This study presents a novel integration of encryption within a federated transfer learning framework for secure classification of renal abnormalities. The proposed framework achieved 92.27% accuracy on the IQ-OTH/NCCD lung cancer dataset, demonstrating its cross-domain generalizability across different medical imaging domains.

A limitation of the proposed framework is that it was evaluated on a single public kidney CT dataset, which may not fully capture the variability seen across real hospital networks. Furthermore, the FL experiments were conducted under an IID data distribution through stratified partitioning, and the performance of the proposed framework under non-IID data distributions was not evaluated. Since real-world healthcare data are often heterogeneous across institutions, further studies are needed to assess the robustness of the framework in non-IID settings. On the privacy side, although AES-256 encryption protects model parameters during transmission, federated learning may still be vulnerable to threats such as gradient inversion and model inversion attacks through shared model updates.

Future work will investigate the performance of the framework under non-IID data distributions and explore the integration of Differential Privacy and Secure Aggregation to strengthen privacy protection. Further efforts will focus on improving communication efficiency, incorporating multimodal medical data sources, and optimizing aggregation strategies to improve scalability, robustness, and broader clinical applicability. Future work will also incorporate explainability techniques such as Grad-CAM and SHAP to enhance clinical transparency and interpretability of the proposed framework.

## Data Availability

The original contributions presented in the study are included in the article/supplementary material, further inquiries can be directed to the corresponding author.
